# Associations between Executive Functions and Sensorimotor Performance in Children at Risk for Learning Disabilities

**DOI:** 10.1155/2023/6676477

**Published:** 2023-09-21

**Authors:** Cecília Tószegi, Andras N. Zsido, Beatrix Lábadi

**Affiliations:** Institute of Psychology, University of Pécs, Pécs, Hungary

## Abstract

Executive functions (EF) and sensorimotor skills play a critical role in children's goal-directed behavior and school readiness. The aim of the current study is to provide new insights into the relationship between executive functions and sensorimotor development by considering the risks associated with learning difficulties. Therefore, we investigate the predictive role of EF and sensorimotor skills in the development of learning difficulties during preschool years. Ninety-five preschool children (5–7 years old) were tested, comparing the performance of children that are at risk of learning difficulties (*n* = 55) to the performance of typically developing children (*n* = 40). Participants completed a battery for the assessment of sensorimotor skills (i.e., Southern California Sensory Integration Test: postural imitation, body midline crossing, bilateral motor coordination, and standing balance with eyes open) and executive functions (i.e., inhibition, cognitive flexibility, and verbal working memory). Our results show that children at risk for learning difficulties exhibited more impairments on sensorimotor and EF measures (inhibition and verbal working memory) when compared with TD children. We ran three separate binary logistic regression analyses to assess the relative influence of EF and sensorimotor functions on predicting risk for learning difficulties. Our findings demonstrated that verbal working memory as EF function (odd ratio (OR) = 0.91, 95% CI 0.78-0.91, *P* = 0.05) and standing balance skills as a sensorimotor skill (odd ratio (OR) = 0.86, 95% CI 0.81-0.98, *P* = 0.01) were the strongest predictors of risk for learning difficulties. The findings point to the importance of supporting children's executive function development and promoting sensorimotor development, as both fundamentally influence school readiness.

## 1. Introduction

### 1.1. Learning Difficulties

Learning difficulties and learning disabilities are clearly defined deficits in academic skill development. Children with learning disabilities [1] are at risk for academic failure (i.e., underachieving, dropping out of primary school) and social-emotional or behavioral problems during their school years [2]. In the educational and psychological literature, the terms *learning difficulties* and *learning disabilities* are often used interchangeably. Indeed, there is considerable overlap in the definition of learning disabilities and learning difficulties, but they have some important distinctions [3]. *Learning disabilities* can be described as specific and diagnosed conditions that exhibit persistent difficulties in developing skills of reading (dyslexia), writing (dysgraphia), or mathematics (dyscalculia) during the formal school years [4]. In contrast, the meaning of *learning difficulties* is broader and is not used for a formal diagnostic category. Learning difficulties refer to the impairment of a learner's ability to perform at a developmentally appropriate academic level. Consequently, we also use the term learning difficulties because our study covers only the early risk factors for academic difficulties in preschool when functional impairment in learning skills cannot be formally tested or diagnosed.

The risk of learning disabilities is detectable before formal literacy and math instruction begin, although learning difficulties are usually identifiable by the time the child enters school. These at-risk children often demonstrate below-average language, literacy, and math skills in kindergarten [5]. At-risk children do not reach age-appropriate developmental milestones at normal levels. During the preschool years, developmental delays are detectable in several domains, including motor, sensorimotor, language, and visual-spatial perception [6–8], but these delays do not manifest in specific symptoms of developmental disorders or show predictive patterns of learning difficulties in primary school.

### 1.2. Associations between Executive Functions, Sensorimotor Performance, and Learning Difficulties

It is well documented that deficits in cognitive processing related to executive functions such as inhibition and self-regulation contribute to children's risk of having learning difficulties during school (e.g., [9–11]). Executive function (EF) is an umbrella term [12] that encompasses three higher-order cognitive processes: response inhibition, working memory, and shifting (cognitive flexibility). These functions collectively support focusing attention, eliminating distractions, adapting quickly, changing circumstances flexibly, regulating drives, delaying reactions, and planning behavior to achieve a specific goal [13]. Children with EF deficits often have difficulties organizing tasks, controlling their emotions, maintaining attention, and regulating their learning in the classroom [1, 14].

Deficits in EF are described in a variety of neurodevelopmental disorders. Dyslexia is the most common form of specific learning disability associated with impairment in EF (in children diagnosed with dyslexia [15], at risk for dyslexia [16]). Previous studies have confirmed that children with dyslexia have problems with central executive skills [17] and retaining visual and auditory information in their working memory [18, 19]. Children with mathematical deficiencies or dyscalculia also have deficits in working memory, response inhibition, processing speed, and cognitive flexibility [20].

Poor sensorimotor integration (motor control) is also typical in specific learning disorders (SLD), developmental dyslexia (DD), and developmental coordination disorder (DCD) [21]. For decades, motor control impairments in children with learning disabilities have been reported [22–24]. These studies showed impairments in posture as well as gross and fine motor skills compared to normally developing peers. A growing body of recent literature supports these findings. For example, a review by Blanchet and Assaiante [25] cited 36 studies reporting on motor impairments in students with SLD. These studies used a variety of qualitative motor tests, which identified minor to severe levels of motor impairment (fine motor, gross motor, and postural skills) in children with SLD. Children and adolescents with SLDs also showed significant impairments in bilateral coordination, balance, and manual dexterity [25]. Furthermore, children with SLD displayed poorer performance in timing precision of motor coordination, demonstrated significant deficits in bimanual tasks, and made more sequential errors [25]. In a recent cross-sectional study of 200 children (100 with SLD and 100 without SLD), researchers assessed motor performance using the second edition of the Bruininks-Oseretsky Test of Motor Skills (BOT-2) [26]. Significant differences were found between children with different types of SLD (dyscalculia, dyslexia, and mixed) when compared to their typical peers in fine motor integration, balance, bilateral coordination, running speed, and agility. Okudaa and Pinheiroa [27] compared the motor performance of students with learning difficulties in relation to students with good academic performance. The students with learning difficulties showed lower performance in fine motor integration, balance, running speed, and agility when compared with their peers [27]. When comparing children with different types of learning disabilities (dyslexia, learning disabilities, learning difficulties, and typical children), Capellini and et al. [28] demonstrated that school-aged children with learning disabilities regardless of the type of impairments performed worse on tests of finger opposition, graphesthesia, and body imitation than typically developing children. Children with dyslexia and learning disabilities performed worse on these tests compared with children with learning difficulties and typically developing children [28].

The relationship between sensorimotor skills and cognitive development was first established by Piaget and Cook [29], who proposed a close link between cognition and sensorimotor functions during development. Sensorimotor and cognitive abilities share specific neural substrates and networks [14], suggesting a link between motor and cognitive development [30–32]. EFs and motor functions follow a similar developmental pathway throughout life [14]. EFs exhibit a longitudinal, inverted U-shaped developmental trajectory, with a dynamic increase from early childhood to early adulthood associated with maturation of the prefrontal cortex, followed by a decline in older adults [13, 33, 34]. Similar to this inverted U-shaped developmental trajectory, the development of various motor skills follows a long maturation over the life course, with early dynamic development peaking in early adulthood [35, 36].

The similar developmental timing of EFs and specific sensorimotor functions has prompted research on the developmental relationship between these functions at different ages [37, 38]. The results of recent studies [39–44] indicated that sensorimotor functions and EFs are inherently intertwined. A study of a large cohort of TD children [43] found a positive relationship between motor performance, visual motor integration, working memory, and fluency. These results showed a parallel development between cognitive and motor functions in 5-6-year-old children. Research has also shown that the development of sensorimotor skills and EFs varies with age [37, 38]. For example, Gordon-Murer et al. [37] found age-related differences in sensorimotor and executive function performance. In children aged 8 to 12 years, better inhibitory control correlated with better accuracy of eye-hand coordination and with higher spatial precision of eye-hand coordination. Higher levels of cognitive flexibility were associated with lower adaptation error and lower variability in proprioception [37]. Better working memory performance is correlated with higher accuracy of eye-hand coordination. However, there was no such association between EF and sensorimotor performance in adolescence [37]. Stuhr et al. [38] also suggest that EF, particularly working memory, and motor function (strength, speed, and dexterity) are linked to the development of preschool children. These studies provide evidence of the interaction between sensorimotor and cognitive functions in children and point to the need to examine the relationship in atypical developmental trajectories where the development of sensorimotor skills and EFs may be impaired [37, 38].

### 1.3. The Present Study

Previous studies showed that children with learning disabilities have low sensorimotor skills and EFs. However, EFs and sensorimotor processing have not been examined together in children at risk for learning difficulties, and it is not clear which component of EFs and sensorimotor skills are the most important predictors of learning difficulties in preschool years. Therefore, the main aim of this study is to identify the relative contributing factors of EFs and sensorimotor skills in the development of learning difficulties and to compare these factors' predictive role in children at risk for learning difficulties.

In the present study, we assess the performance of sensorimotor skills and EFs performance in a group of preschool children screened out at risk for learning difficulties (RLD) and a control group (typically developing children (TD)) matched for age, sex, and maternal education. Based on the previous literature (see above), we expected the poor performance of inhibition and working memory (components of EFs) along with bilateral motor coordination and standing balance (components of sensorimotor skills) are the factors that predicted the risk of learning difficulties in children. Importantly, our study also involved IQ to examine its effect on learning difficulties, which has not been done in previous research to date. Such research is important to identify factors related to the risk of learning difficulties in preschool and develop specific intervention programs for children at risk before starting school.

## 2. Method

### 2.1. Participants and Procedure

The final sample consisted of 95 children divided into two groups: 55 children at risk for learning difficulties (RLD) and 40 children with typical development (TD) aged 5 to 7 years (*M* = 71.5, months SD = 5.9). Children in the RLD group were recruited specifically from the Educational Service of Baranya County, Hungary (the National Diagnostic and Therapeutic Institutional Network), and typically developing children (TD) were recruited from three local kindergartens.

The inclusion criteria for admission of children at risk of learning difficulties are based on the complex psychological and educational assessment protocol of the Educational Service of Hungary. The assessment examines preschool children before starting school to screen the risk of learning disabilities and provide an immediate intervention program. Based on this protocol, children are considered at risk for learning difficulties if they have a developmental delay in at least one of the following domains: elementary literacy, numeracy, language skills, visual perception, gross and fine motor skills, and social-personal maturity. The assessment data of children collected by the educational services were not obtained for research. Children in the TD (control) group were selected from three local kindergartens and were matched to the RLD group by age, gender, and maternal education. Exclusion criteria for both groups were prematurity, developmental disorders (such as ASD, ADHD, DCD, and DLD), genetic disorders, severe sensory impairment (i.e., blind or deaf), and low IQ scores (IQ < 85). The data collection was preceded by written information and consent requests from heads of institutions and participants' parents. The information and consent of the children were obtained orally. Parents and children volunteered to participate in the study. Ethical approval for the study was obtained from the Hungarian United Ethical Review Committee for Research in Psychology (reference number: 2018/96).

Parents completed a survey about demographic information and the medical history of their children. The breakdown of the demographic data by each group can be found in Table 1. Each child was tested individually by a trained research assistant (RA) in a quiet room. First, the children completed a series of executive functions and then sensorimotor tasks administered by trained research assistants. Children completed the tasks in two sessions with a 20-minute break and received a small reward at the end of each session.

### 2.2. Measures

#### 2.2.1. Demographics and Health Information

Parents completed demographic and health-related questions addressing children's age, sex, and mother's education. The mother's highest level of education (classified by college or university, high school, vocational school or below) was used as an indicator of socioeconomic status (SES). Health information included gestational age at birth, birth weight, and health status (e.g., brain injury, medical complications and chronic diseases affecting child development, and developmental disorders and medical diagnosis). Psychological assessment of children in the RLD group included full-scale IQ scores (the fourth edition of the Wechsler Preschool and Primary Scale of Intelligence (WPPSI-IV) [45]). TD children's intelligence was measured using the Raven Color Progressive Matrices ((RCPM) [46]) because there was no option to assess the full WPPSI-IV. The scores of WPPSI-IV and RCPM were converted to standard scores with a mean of 100.

#### 2.2.2. Executive Functions

Children completed a performance-based battery of three tasks to test different aspects of executive function: response inhibition, cognitive flexibility, and verbal working memory.

The *response inhibition* component of executive functions was measured with a *Go/NoGo-type task.* The task was programmed using PsychoPy software [47] following the age-appropriate protocol adapted for use with preschool children [48]. The Go/NoGo task required children to make a response (by pressing the space bar) to each appearance of a fish (“Catch the fish!”) on a computer screen on Go trials but to inhibit the response when a shark appeared on the screen (“Let's not catch the shark!”) on NoGo trials. Prior to the task, children received instruction and practice trials with 10 trials (8 Go, 2 NoGo trials). The experimental task consisted of 75 trials (80% Go, 20% NoGo) divided into three pseudorandom blocks. The blocks were separated by a short pause. The Go and NoGo trials were presented in pseudorandom order, and a block never started with a NoGo trial. Each stimulus was presented on screen for 1500 ms separated by 1000 ms interstimulus intervals. Performance with more than 30% inaccuracy on Go trials was excluded from the complete blocks. We also removed the extremely fast-responding trials (<300 ms) because children may not have responded to the stimulus. We calculated proportional accuracy on Go and No/Go trials, representing the percentage of correct responses.

The *cognitive flexibility* component of executive functions was measured using the *Dimensional Change Card Sort* (DCCS) task. We applied this task following the protocol of a previous study [49]. This task is a widely used measure of cognitive flexibility that has been adapted for use with preschool children. Two target cards are presented that differ in two dimensions (shape and color; e.g., a blue rabbit and a red boat). Children must match the cards (3 red rabbits and 3 blue boats) to one of two targets (shape or color). “Switch” trials were also conducted, in which children were asked to change the dimension being sorted (e.g., children sorted cards that matched the shape, then children were asked to match the cards on color on the next trial). The procedure followed the 4 steps of the standard test version: demonstration and practice trials (2 trials), preswitch phase (6 trials), postswitch phase (6 trials), and border test version (12 trials). After the demonstration and practice trials, children first sorted the cards by one of the two targets (e.g., color, 6 trials), then the rule was changed (postswitch phase), and they sorted the cards by the other target (e.g., shape, 6 trials). The order in which the rules (color and shape) were presented was counterbalanced across children. If a child solved at least 5 of the 6 trials before and after the switch, then he/she could proceed immediately to the advanced version (i.e., border test) of the task. In this version, children must sort the cards with the black border by color and the cards without the border by shape. Scores were determined based on the percentage of correct responses (correctly sorted cards) for preswitch, postswitch, and border trials. Reaction time was also coded based on video footage.

We measured *verbal working memory* (and language competence) using the Hungarian Sentence Repetition Test. This test measures syntactic development through the accurate, immediate repetition of sentences of increasing length and varying structural complexity in children aged 4-6 years [50]. The test consisted of 10 sentences of increasing complexity (see examples in Table 2). The sentences were between 8 and 15 syllables long. The indicator of performance on the test is the number of correctly repeated sentences. The increasing length and complexity of the sentences allow the measurement of both scope and operational load [51].

#### 2.2.3. Sensory Processing

To examine the children's sensorimotor performance, we used the subtests of the Southern California Sensory Integration Test (SCSIT) that were relevant to the present study: posture imitation, crossing the body midline, bilateral motor coordination, and standing balance with eyes open [52]. The SCSIT is a standardized test used to assess the sensory integration process. In selecting an appropriate instrument for measurement, we considered that the cultural independence of the SCSIT has been demonstrated in previous national research [53]. Following the procedure of this previous research, we calculated *z*-scores for the subtests of posture imitation, body midline crossing, and bilateral motor coordination using age norms.

In the *posture imitation task*, the child imitates the mirror image of the examiner's posture (instruction: “You will do the same thing with your arms and hands as I show you. Let's see how fast you can imitate.”). After children successfully imitated the gait posture, the test includes the demonstration of 12 other postures. To ensure fair scoring, the imitated postures were recorded in photographs. Scoring of the postures was based on the following criteria: an accurate imitation within 3 seconds resulted in 2 points; within 4-10 seconds was 1 point; and failure to imitate or timeout resulted in 0 points.

In the *body midline crossing* task, the examiner was seated facing the child. The child is asked to imitate the touching of the ears or eyes with the hand. First, 4 practice items were presented. Each position was held until the child responds. During the practice, the examiner assisted in the imitation with explanations and by moving the child's hands if necessary. After the practice, no further assistance is given to the child. A series of eight items was presented three times. Imitation involved an equal ratio of homolateral and midline crossing movements. Scoring is based on success in crossing the midline. Each correct imitation was awarded 2 points; each incorrectly started but corrected imitation was 1 point; and each unsuccessful imitation was 0 points.

In the *bilateral motor coordination* test, the main difference compared to the body midline crossing test was that the child was not allowed to imitate until the demonstration task was been completed. The behavioral dimension tested is the integrated ability to move the two upper limbs together. Coordination is assessed by the timing and continuity of the interaction of the two hands. The items consist of movement patterns or sequences that were repeated once or twice. Scoring was similar to that for the body midline crossing test.

In the *standing balance with eyes opened task,* the child was asked to stand on one leg in a place where he or she could not lean. The child put the backs of their hands under their arms and placed their hands on their chest. Once the child lifted their foot, we started to measure the time until the child put their foot down, even for a moment, extended their arm to balance, started jumping, or moved their foot. If the child immediately lost their balance when he/she lifted the leg, we repeated the test. The point value was equal to the time spent in balance, expressed in seconds. The measurement was determined for each leg, and the score was obtained by the mean value.

### 2.3. Data Analysis

To ensure that only valid data were included in the analyses, we first identified invalid data. Missing data occurred as a result of participants' technical failure or tiredness to complete the task. There are missing data in the Go/NoGo (*N* = 5), the bilateral coordination (*N* = 1), sentence repetition (*N* = 6), and the DCCS (*N* = 2) tasks.

Data analysis was conducted using the IBM SPSS Statistics 21 software. Significance was set at *P* ≤ .05. The data distribution failed the Shapiro–Wilk test of normality; therefore, we used nonparametric Mann–Whitney *U* tests to examine group differences. First, we analyzed the possible differences in demographic variables between the RLD and TD groups. Then, we analyzed the descriptive differences in performance of sensorimotor skills (postural imitation, body midline crossing, bilateral motor coordination, standing balance eyes open, and standing balance eyes closed) and EFs (response inhibition, cognitive flexibility, and sentence repetition) between the groups. In the main analysis, we carried out three binary logistic regression analyses to examine the relative influence of EF subfactors and the different sensorimotor functions predicting status (and risk/no risk as the dependent variable). In the first two models, we separately tested the role of EF components and sensorimotor components. Finally, we include both EFs and sensorimotor functions as predictive variables (and risk/no risk as the dependent variable); furthermore, we also include IQ and age to evaluate their contributing effect to the risk of learning difficulties.

## 3. Results

### 3.1. Demographics

As Table 1 shows, there were no significant differences between TD and children at risk for learning difficulties regarding age, gestational age, birth weight, and maternal education. However, there was a significant difference in the proportion of children based on gender (*X*^2^ = 13.4, *P* < .001); more males were involved in the TD group.

### 3.2. Sensorimotor Skills

See the exact statistical results and descriptive in Table 3. As expected, children in the RLD group scored significantly lower than those in the TD group on all tasks measuring sensorimotor abilities including postural imitation, body midline crossing, bilateral motor coordination, and standing balance with eyes open.

### 3.3. Executive Function

Exact statistical results and descriptives are shown in Table 4. In line with our hypotheses, we found a significant difference between the performance of children in the RLD compared to the TD group on the following EF tasks: Go trials, NoGo trials, and the sentence repetition task. Contrary to what was expected, we did not find any significant differences in the accuracy rate of the card-sorting task. However, the mean reaction time was significantly higher in the RLD group in both the preswitch and postswitch blocks compared to the TD group. There was no significant difference between the groups in the border task version.

### 3.4. Binary Logistic Regression Analysis

The findings presented above report the presence of significant differences in sensorimotor abilities and EF between TD and RLD children (except in the card sort task); however, it is not certain that these tasks can successfully discriminate between subjects. Therefore, we ran three binary logistic regression analyses to evaluate the relative influence of EF subfactors and the different sensorimotor functions predicting status, i.e., at risk or not at risk for learning difficulties. The first model tested the effects of EF subfactors, while the second model tested the effects of sensorimotor functions on the risk for learning difficulties status as a dichotomized dependent variable (risk = 1; no risk = 0). Finally, we carried out a third regression analysis including both EFs and sensorimotor functions as predictive variables (and risk/no risk as the dependent variable). As we wanted to ensure that children's IQ, and age were not contributing to the differences found between the groups, therefore, we included these variables in the analysis as well.

In the first regression model (see Table 5 for exact statistical results), we used separately the performance of executive functioning tasks (total accuracy rate of card sort, Go trials, NoGo trials, and sentence repetition) as predictors. The overall model for the risk of learning difficulties was significant (*χ*^2^(4) = 33.4, *P* < .001) and presented an adequate adjustment value (Cox-Snell *R*^2^ = .33, Nagelkerke *R*^2^ = .44). The model correctly classified 73.5% of the cases patients (72.9% of the risk participants and 74.3% of the no-risk participants were correctly classified). Results indicated that subjects were more likely to be at risk of learning difficulties if their performance was poorer in sentence repetition task and NoGo trials. The Go trials and card sort scores were nonsignificant.

The second regression analysis (see Table 6 for detailed statistical results) evaluated the contribution of sensorimotor skills to the risk of learning difficulties. The model was also significant (*χ*^2^ (4) = 54.7, *P* < .001) and presented a good adjustment value (Cox-Snell *R*^2^ = .41, Nagelkerke *R*^2^ = 0.59). The model correctly classified 78.7% of the cases (85.2% of risk status and 70.0% of no risk status were correctly classified). Specifically, the performance of bilateral coordination and standing balance predicted the classification of participants into the learning difficulties group. The effect of postural imitation and midline crossing was nonsignificant.

The third overall regression model was used to evaluate the relative influence of EF and sensorimotor functions in predicting the status of risk for learning difficulties. The additional impact of children's IQ and age was also tested in this analysis.  Table 7 shows the detailed statistical results. The overall model was significant (*χ*^2^ (9) = 64.6, *P* < .001, Cox-Snell *R*^2^ = .60, Nagelkerke *R*^2^ = .82). The model correctly classified 93% of the cases (84% of no-risk status and 97.8% of risk status were correctly classified). Significant predictors were the performance of sentence repetition task and standing balance with eyes open. Other EF and sensorimotor measures, and the children's general demographic data did not emerge as significant predictors.

## 4. Discussion

Previous studies demonstrated a relationship between EFs and sensorimotor performance in childhood [37, 43] and found specific impairments in EF and sensorimotor function in children with learning disabilities. However, there is no study that investigated the EFs and sensorimotor skills together as early predictors of learning disabilities in preschool-aged children. Therefore, we tested which components of EFs and sensorimotor skills predicted whether children would be identified as at risk for learning difficulties.

The major finding of our study is that verbal working memory and standing balance play a leading role in predicting the risk of learning disability. Other components of EF and sensorimotor skills as well as IQ did not contribute significantly more to prediction. In our model, standing balance was the most influential variable; its poor performance increases the risk of learning disabilities. This result is consistent with previous studies noted in the introduction, showing that impairment in standing balance is related to the risk for developmental dyslexia [54–57] and learning disabilities [21, 26]. Balance mechanisms are important during development because they allow us to maintain a positional equilibrium by coordinating internal and external forces on the body while using sensory information, such as visual inputs, vestibular inputs, and proprioceptive inputs [58]. In childhood, visual information provides stronger input to balance than proprioceptive information, and between four and six years of age, balance mechanism undergoes a change, with the dominance of visual input gradually being replaced by proprioceptive information [58]. It seems that RLD children are more vulnerable to balance mechanisms in childhood, which may have implications for their learning abilities. For instance, RLD children might have difficulty using visual information to calibrate postural control of balance. Barela et al. [54] found that children with developmental dyslexia oscillated more than nondyslexic children in both stationary and oscillating balance conditions. Furthermore, the connection between visual information and body sway is weaker and more variable in dyslexic children, indicating that dyslexic children have difficulty using visual information to calibrate control of balance [25, 54, 59]. Poor sensorimotor integration is also known in children with specific learning disorders, developmental dyslexia, and developmental coordination disorder [21]. Our results also show that besides the standing balance, bilateral motor coordination is a sensitive indicator of learning difficulties which is consistent with previous studies [6, 7]. Children with RLD showed weaker sensorimotor performance, including bilateral motor coordination [60] and standing balance. These results underscore the idea that there is a positive and significant relationship between sensorimotor skills and academic performance characterized by cognitive skills. Surprisingly, the performance of postural imitation was not a significant predictor in the regression models; however, the descriptive group comparison showed that RLD children performed worse than TD children. Prior studies [61, 62] investigated the imitation of complex, novel postures, and gesture sequences in children with and without probable developmental coordination disorder using the postural practice and sequence practice subtests of the Sensory Integration and Praxis Tests [63]. Children at risk for developmental coordination disorders were less accurate in imitation tasks than control children.

Verbal working memory was the other influential variable in our final model, and poorer performance of WM predicted a higher risk of learning difficulties. Many studies have documented that WM deficits contribute to different types of learning difficulties. A meta-analysis of working memory deficits in children with learning difficulties [64] found that all types of difficulty groups demonstrated deficits in verbal WM and numerical WM, and children with reading difficulties showed the most severe WM impairments. Our results are in line with research demonstrating the involvement of working memory, cognitive flexibility, and response inhibition in children with learning difficulties and learning disabilities. For instance, children with dyslexia [16] and children at risk of dyslexia [65] showed deficits in WM. Preschool children who performed poorly in numeracy or mathematics also showed lower scores in all three EF domains [20]. Similarly, EF has a major predictive role in numeracy development [66]. According to the classic model of Baddeley [67, 68], verbal WM is responsible for the phonological loop, which is coordinated by the central executive and they direct attention to relevant information. Furthermore, WM is a good predictor of general cognitive functioning, learning processes [69], and academic performance [70]. Therefore, problem of the central executive, including WM (phonological loop and visuospatial sketchpad), is a common cause of learning difficulties [64].

Regarding the other performance-based executive function, the predictive role of response inhibition was not confirmed by the final regression analysis when all predictor variables were included. However, it was a significant predictor for the risk of learning disabilities in the model when we tested only the effects of executive function separately. These results showed that using the Go/NoGo paradigm, a lower performance level, and more errors in NoGo trials increased the risk for learning difficulties. Indeed, in the early childhood literature, many measures of EF have been shown to predict academic skills (e.g., [71]), and deficits in inhibition and planning predict learning disabilities [72]. However, mixed results have been found for inhibition deficits in children with learning disabilities [73]. A meta-analysis [74] demonstrated the relationship between cognitive flexibility (shifting) and specific learning disabilities (reading and math performance), but in our study, the shifting was also not a predictor of the risk of learning disabilities. Cognitive flexibility relies on the development of working memory and inhibitory control and emerges much later in development [13]. To switch perspectives during cognitive flexibility, one needs to activate a different perspective in one's working memory and inhibit the previous perspective. The cost of switching to a new task can be reflected in performance in two ways: the number of errors increases and responses are typically slower during the new task [75]. The majority of children are able to switch on the DCCS task by age 4.5 to 5 years on the sorting dimensions [13, 49]. Accordingly, we found no difference in the accuracy of task performance following switching between the RLD and TD groups. However, the switch proved to be more costly among RLD children. Based on the descriptive data, significantly slower reaction times were found in the postswitch phase among RLD children compared to TD children.

The present study is not all-encompassing, and future research needs to examine the executive function and sensorimotor skills involving a larger sample by refining the measurement. A larger sample size would also allow us to differentiate the group in terms of risk of specific learning disability for dyscalculia and dyslexia. Longitudinal assessment of developmental risk would increase the possibility of a more effective design of prevention programs. A further limitation is that the Dimensional Change Card Sort (DCCS) task did not support to be an appropriate measure to distinguish TD from at-risk children in the 5-7 age group. A ceiling effect was observed in the postswitching task, while the border test version was challenging for both groups of children. Further research on cognitive flexibility in children with RLD will help to clarify its role in learning difficulties. Specific deficits in working memory also remain unclear. Previous literature found mixed results regarding the involvement of the verbal and visuospatial WM, but our study did not investigate the visuospatial WM at all.

## 5. Conclusions

In conclusion, to our knowledge, this is the first study to investigate the relative contribution of cognitive and sensorimotor development in learning difficulties for preschool-age children. A strong relationship has been shown in preschool age children, particularly in working memory and sensorimotor function [37, 38, 40]. Our results show that sensorimotor (bilateral coordination, balance) and executive functions (working memory, response inhibition) separately predict developmental risk in preschool children, while the model with the strongest predictive value is the one in which sensorimotor and executive functions are combined. It appears that working memory and balance play a key role in overall cognitive-motor functioning at the age of 5-6 years [37, 38, 56]. Our results highlight the importance of supporting children's executive function development and enhancing their sensorimotor development. All these results may have practical implications for the design of intervention programs, as both executive function and sensorimotor skills are key determinants of school readiness. The results of impact evaluations of intervention programs also support the case for early development and preventive programs to compensate for disadvantages [48] in the domains of these functions.

## Figures and Tables

**Table 1:** Demographic characteristics of children at risk for learning difficulties compared to typically developing children.

	Typically developing children (*N* = 40)	Children at risk for learning difficulties (*N* = 55)	*t* or *X*^2^
	*N* (%) or *M* ± SD	*N* (%) or *M* ± SD	
Child's age (months)	71.4 (7.53)	71.6 (6.30)	-.01
Child's gender—female	26 (65)	15 (27.2)	14.7^∗∗∗^
Maternal education			.75
College or above	8 (20.0)	14 (25.9)	
High school	11(27.5)	17 (31.5)	
Vocational school or below	21 (52.5)	23 (42.5)	
Gestational age (weeks)	39.3 (1.35)	39.2 (1.11)	.10
Birth weight (g)	3314.3 (503.26)	3346.6 (367.3)	-.33
Child's IQ	106.5 (11.87)	92.8 (9.18)	6.34^∗∗∗^

Note: ^∗^*P* < 0.05; ^∗∗^*P* < 0.01; ^∗∗∗^*P* < 0.001.

**Table 2:** Example items from the sentence repetition task.

Item no.	English translation	Original Hungarian
1.	The train driver likes pancakes.	A mozdonyvezető szereti a palacsintát.
2.	Elephants like bananas that are sweet.	Az elefánt azt a banánt szereti, ami édes.
3.	The soldier who drives the car is brave.	A katona, aki vezeti a kocsit, az bátor.

**Table 3:** Statistical results and descriptive statistics regarding the differences between typically developing children (TD) and children at risk for learning difficulties (RLD) in terms of sensorimotor (SM) scores on the four relevant tasks.

SM task	TD children (*N* = 40)	RLD children (*N* = 55)	Mann–Whitney *U* (df = 86)	*P*
	M (SD)	M (SD)		
Postural imitation	16.63 (4.14)	11.22 (4.54)	430	<.001
Midline crossing	18.00 (5.60)	13.95 (6.12)	656	<.001
Bilateral motor coordination	11.50 (4.08)	6.43 (4.11)	411	<.001
Standing balance with eyes open	29.10 (17.54)	10.93 (7.64)	336	<.001

**Table 4:** Statistical results and descriptive statistics regarding the differences between typically developing children (TD) and children at risk for learning difficulties (RLD) in terms of executive function (EF) tasks.

	TD children M (SD)	RLD children M (SD)	Mann–Whitney U df	*P*
EF tasks				
Go/Nogo			df = 90	
Go	.95 (.06)	.92 (.06)	610	.002
NoGo	.90 (1.10)	.82 (.15)	672	.009
Card sort			df = 92	
Total	.85 (2.88)	.82 (2.93)	925	.313
Preswitch^∗^	1.0 (.0)	.98 (0.13)		
Postswitch^∗^	1.0 (.0)	1.0 (.0)		
Border phase	.71 (.24)	.67 (.22)	937	.362
Mean RI preswitch	2.35 (.82)	2.95 (1.24)	696	.005
Mean RI postswitch	2.24 (.90)	2.80 (1.18)	682	.004
Mean RI border phase	4.46 (1.88)	4.59 (1.46)	919	.297
Sentence repetition	.84 (.12)	.57 (.28)	df = 89 397	<.001

Note: ^∗^Mann-Whiney U was not available because of the ceiling effect.

**Table 5:** The results of the binary logistic regression predicting the likelihood of the risk of learning difficulties status (risk = 1; no risk = 0) with the performance on executive functioning tasks as predictors.

EF task	*P* value	OR	95% CI
Go	.70	.98	.922 to 1.056
NoGo	.024	.95	.912 to .994
Card sort	.58	.98	.944 to 1.033
WM sentence repetition	<.001	.95	.911 to .971

Note: the full model was significant (*χ*^2^ (4) = 33.4, *P* < 0.001, Cox-Snell *R*^2^ = 0.33, Nagelkerke's *R*^2^ = 0.44).

**Table 6:** The results of the binary logistic regression predicting the likelihood of the risk of learning difficulties status (risk = 1; no risk = 0) with performance on sensorimotor (SM) tasks as predictors.

Sensorimotor tasks	*P* value	OR	95% CI
Postural imitation	.13	.89	.765 to 1.035
Midline crossing	.82	1.01	.911 to 1.24
Bilateral motor coordination	.019	.83	.722 to .971
Standing balance	.001	.89	.832 to .952

*Note:* the model was significant (*χ*^2^ (4) = 54.7, *P* < .001, Cox-Snell *R*^2^ = .41, Nagelkerke's *R*^2^ = .59).

**Table 7:** The results of the binary logistic regression predicting the likelihood of the risk of learning difficulties status (risk = 1; no risk = 0) with executive function (EF) measures, sensorimotor (SM) skills, IQ, and age as predictors.

	*P* value	OR	95% CI
EF tasks			
NoGo	.33	1.01	.40 to 2.53
Card sort	.47	.83	.51 to 1.38
WM sentence repetition	.05	.91	.78 to .99
SM tasks			
Postural imitation	.08	.82	.66 to 1.02
Midline crossing	.82	.98	.83 to 1.15
Bilateral motor coordination	.74	.96	.78 to 1.84
Standing balance	.01	.86	.81 to .98
IQ	.11	.92	.83 to 1.02
Age	.51	1.11	.88 to 1.30

Note: the full model was significant (*χ*^2^ (9) = 64.6, *P* < .001, Cox-Snell *R*^2^ = .60, Nagelkerke *R*^2^ = .82).

## Data Availability

The data used to support the findings of this study are available from the corresponding author upon request.
